# Slowly Expanding Lesions in Multiple Sclerosis: A Systematic Review and Meta-Analysis

**DOI:** 10.3390/neurosci7020034

**Published:** 2026-03-06

**Authors:** Mohammad Yazdan Panah, Mehra Fekri, Zahra Zahedi, Hossein Bagheri, Saeed Vaheb, Farhad Mahmoudi, Vahid Shaygannejad, Omid Mirmosayyeb

**Affiliations:** 1 Isfahan Neurosciences Research Center, Isfahan University of Medical Sciences, Isfahan 81839-83434, Iran; mohamad.yazdanpanahh@gmail.com (M.Y.P.); fekrimehra80@gmail.com (M.F.); zahedi.79.zahra@gmail.com (Z.Z.); parsabagheri40@gmail.com (H.B.); saeedvaheb.sv@gmail.com (S.V.); v.shaygannejad@gmail.com (V.S.); 2 Department of Neurology, University of Miami, Miami, FL 33136, USA; fxm707@miami.edu; 3 Department of Neurology, Isfahan University of Medical Sciences, Isfahan 81746-73461, Iran

**Keywords:** multiple sclerosis, slowly expanding lesion, magnetic resonance imaging

## Abstract

Background: Slowly expanding lesions (SELs) have been introduced as a radiological marker of chronic active demyelination and smoldering inflammation. These lesions are recognized as indicators of disability worsening and brain atrophy in people with multiple sclerosis (PwMS). We aimed to provide an overview of the available evidence on the prevalence and clinical relevance of SELs in PwMS. Methods: PubMed, Embase, Scopus, and Web of Science were systematically searched up to 25 May 2025, to identify studies evaluating SELs in PwMS. Risk of bias was assessed using the Newcastle–Ottawa Scale. We conducted a thorough review to evaluate the clinical relevance of SELs in MS. Additionally, a meta-analysis was performed using R software to estimate the pooled prevalence of SELs in MS. Results: Twenty studies on 4970 PwMS met the inclusion criteria. Meta-analysis indicated that the pooled prevalence of SELs in PwMS was 57.1% (95% CI: 44.9% to 69.3%). Moreover, the systematic review showed that SELs were associated with chronic neuroinflammation, ongoing demyelination, disability, microstructural damage, and axonal degeneration. Intervention studies also indicated that the number and volume of SELs were decreased following the administration of disease-modifying therapies. Conclusions: SELs are revealed to affect around half of PwMS and are associated with disability and disease progression in MS. These results highlight the potential role of SELs as a critical radiomarker in MS. However, future studies are warranted to validate these preliminary findings.

## 1. Introduction

Multiple sclerosis (MS) is a chronic autoimmune disease that targets the central nervous system (CNS) and causes multifocal inflammation, demyelinating lesions, and axonal loss. It affects more than two million people around the world, with women being affected more frequently than men [1,2]. The MS diagnostic criteria have been recently revised, and according to the 2024 McDonald criteria, the diagnosis of MS is based on a combination of clinical features, CSF (cerebrospinal fluid) biomarkers, and ruling out diseases that mimic MS. Brain and spinal cord magnetic resonance imaging (MRI) is the most useful modality for confirming and monitoring CNS demyelinating lesions [3,4]. Conventional MRI techniques can reveal white matter lesions (WMLs) on T2- and T1-weighted images and gadolinium enhancement images during inflammation [5]. Acute lesions are synonymous with transient blood–brain barrier disruption and are widely used as surrogate indicators of disease activity in clinical practice in MS [6,7].

Following the acute relapse, MS lesions may undergo some pathological processes, as they become quiescent, remyelinate (shadow plaques), or remain chronically active and form chronic active lesions (CALs) [8,9]. CALs are a key pathological feature in PwMS, particularly in progressive MS (PMS). These lesions contain a demyelinated core with axonal loss and an inflammatory rim composed of activated microglia and macrophages. The rims can be iron-laden, and perivascular B cells and T cells may also be present [10].

Two MRI-visible subtypes of CALs are important markers of this smoldering inflammation, including paramagnetic rim lesions (PRLs) and slowly expanding lesions (SELs) [11]. PRLs, visible on susceptibility-based MRI, are used as imaging markers of chronic active inflammation in MS. They have been associated with progressive disease course and worse long-term outcomes, and have been considered in the 2024 revised McDonald diagnostic criteria [3,12]. SELs manifest longitudinally on MRI as non-enhancing lesions that progressively enlarge over time, representing chronic demyelination and axonal loss [13].

SELs are considered key markers for diagnosing and monitoring disease progression in MS [14]. Notably, the presence of more SELs is associated with brain atrophy, increased disability, and a poorer response to treatment in MS [14,15]. As SELs provide valuable insights into developing therapeutic approaches to address the neurodegenerative processes underlying MS, evaluating their characteristics is critical. SELs may present a promising approach to preventing chronic disability and enhancing patient outcomes through early detection and treatment strategies [16,17,18].

Research on SELs in MS has increased recently. However, variations in study design, patient cohorts, and imaging methodologies led to inconclusive evidence [19]. These variations prevent forming direct inferences about the role of SELs as markers of chronic demyelination and disease progression in MS. Therefore, this review aimed to estimate the pooled prevalence and clinical relevance of SELs among PwMS.

## 2. Methods

This systematic review and meta-analysis was performed based on the PRISMA guidelines [20]. The protocol of this review was prospectively registered in PROSPERO (CRD420251163408).

### 2.1. Search Strategy

PubMed, Embase, Scopus, and Web of Science were comprehensively searched up to 25 May 2025. The search strategy included keywords related to “multiple sclerosis” and “slowly expanding lesions,” and their corresponding MeSH terms. Google Scholar and the reference lists of the included studies were manually searched to find more relevant studies. A detailed search strategy is presented in the Supplementary Material, Table S1.

### 2.2. Study Selection

All search results were imported into EndNote X21, and duplicates were removed. Two independent reviewers (ZZ and HB) screened the titles and abstracts to eliminate studies that did not meet the inclusion criteria. Then, the full texts of potentially relevant articles were thoroughly reviewed. When any disagreement appeared, consensus was achieved through consultation with the third reviewer (OM).

### 2.3. Eligibility Criteria

Studies with the following criteria were considered eligible: (1) published in peer-reviewed journals; (2) written in English; (3) used observational designs, including cross-sectional, cohort, and case–control studies and clinical trials with relevant baseline data; (4) included adult participants (age more than 18 years); (5) involved individuals with a confirmed diagnosis of MS based on established diagnostic criteria; and (6) assessed or identified SELs in PwMS using MRI-based approaches, including longitudinal imaging analysis supported by dedicated lesion-analysis software or validated in-house computational methods. Studies were excluded with any of the following conditions: (1) publications in languages other than English; (2) reviews, preprints, institutional repositories, case reports, letters, or conference abstracts; (3) preclinical studies, including in vitro experiments and in vivo animal studies; (4) inclusion of participants with other neurological disorders that might interfere with the evaluation of SELs; or (5) insufficient data availability, even after contacting the corresponding authors for clarification or additional information.

### 2.4. Data Extraction

Data were independently extracted from the included studies by two reviewers (ZZ and HB). Any disagreements during this process were resolved by consulting the third reviewer (OM). Information, including study characteristics (first author, publication year, country, and design), participant demographics (sample size, gender ratio, and age), clinical features (MS subtype, EDSS score, and disease duration), MRI parameters (scanner type, analysis software, SEL definition, magnetic field strength, and image analysis software), and major findings of the study were extracted. Also, in longitudinal studies and RCTs where data were reported at multiple follow-up time points, we extracted baseline data for all relevant outcomes.

Moreover, the following information on the SEL assessment methodology was sought: the software used for MRI analysis (e.g., Jim 7 and 9, FreeSurfer 7.1.1, ANTs, Jazz, or in-house software) and the quantitative criteria used for lesion assessment.

In general, SEL assessment in all studies was based on longitudinal MRI of lesion growth. It entailed one or more of the following parameters: (i) the number of SELs per subject, (ii) the spatial growth or volumetric enlargement of individual lesions, and (iii) total SEL burden as the cumulative volume or surface area of all detected SELs. When available, automated or semi-automated software was used to monitor lesion radial growth and measure lesion enlargement between paired scans.

### 2.5. Risk of Bias Assessment

The methodological quality and risk of bias (ROB) of the included observational studies were assessed using the Newcastle–Ottawa Scale (NOS) [21], with a maximum score of 10 for cross-sectional studies. Cross-sectional studies scoring 7 to 10 were considered good quality (low risk), scoring 5 to 6 were satisfactory quality (moderate risk), and scoring 0 to 4 were unsatisfactory quality (high risk). ROB of the included studies was independently assessed by two reviewers (MF and SV). Any uncertainty or disagreement was resolved by involving the third reviewer (OM).

### 2.6. Data Analysis

Data analysis was performed using R software version 4.4.0 [22]. Data were summarized using descriptive statistics and expressed as frequencies and percentages. A meta-analysis was conducted to estimate the pooled prevalence of SELs among PwMS, and the findings were illustrated through forest plots. Regarding duplicate cohorts, those with the most comprehensive data were included in the meta-analysis. Considering the methodological heterogeneity among the included studies, we used a random-effects model to analyze the proportions on the logit scale and report results with 95% confidence intervals (CIs). Statistical heterogeneity among studies was evaluated using the χ^2^ test and quantified with the I^2^ statistic [23]. Publication bias or small-study effect was detected using Begg’s and Egger’s tests [24,25]. The statistical significance threshold for all analyses was *p* < 0.05.

## 3. Results

### 3.1. Literature Search and Study Selection

**Figure 1:**
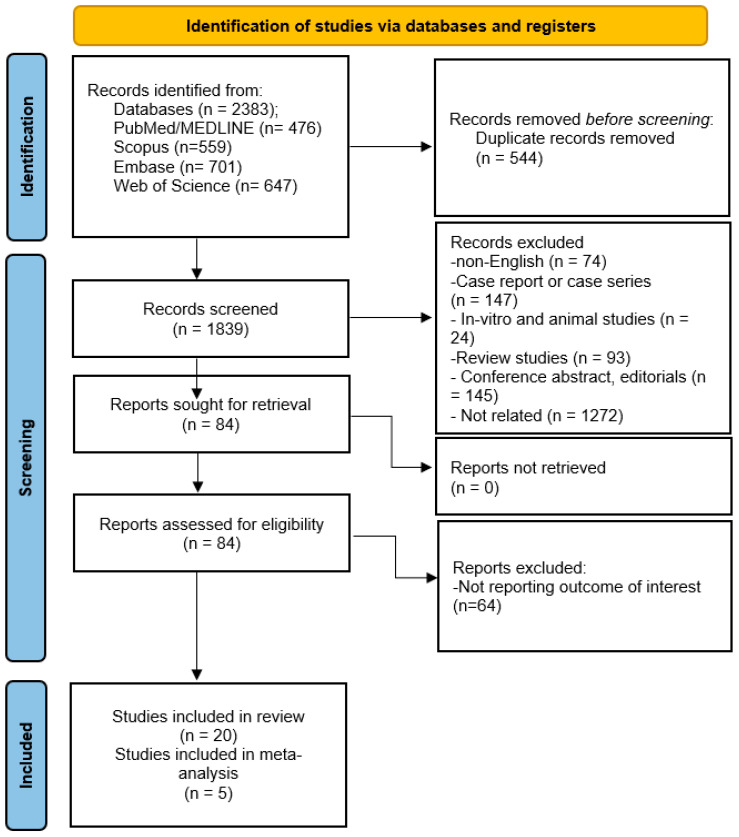
PRISMA 2020 flow diagram of the study process.

The database search initially yielded 2132 records. Duplicate removal was performed using EndNote X21, after which 1683 articles remained for screening. Title and abstract evaluation were conducted manually according to predefined inclusion and exclusion criteria, excluding irrelevant studies and leaving 84 articles for full-text assessment. Full-text review was also performed manually, resulting in the inclusion of 20 studies in the systematic review, of which five were incorporated into the meta-analysis (Figure 1).

### 3.2. Characteristics of the Included Studies

The systematic review incorporated a total of 20 studies published between 2016 and 2025, covering a wide range of study designs, including cohorts, cross-sectional, and randomized controlled trials. The included studies enrolled diverse populations across multiple countries, such as Canada (*n* = 5) [19,26,27,28,29], the United Kingdom (*n* = 5) [18,30,31,32,33], the United States (*n* = 5) [17,34,35,36,37], Italy (*n* = 2) [38,39], Japan (*n* = 1) [40], Switzerland (*n* = 1) [41], and Australia (*n* = 1) [42]. Participants in the included studies were 4970 PwMS (61.4% female) with a mean (SD) age of 42.9 (10.6) years, an EDSS of 3.6 (1.9), and a disease duration of 7.8 (6.7) years. All studies employed high-field MRI, with scanner strengths ranging from 1.5 to 7 Tesla (most commonly 3 Tesla), and they utilized various image analysis software. Jim software was commonly used for lesion analysis, whereas others applied FreeSurfer, Advanced Normalization Tools, or relied on in-house/automated methods. The operational definition of “slowly expanding lesion” varied between studies; many defined SELs qualitatively as chronic T2 lesions that expand radially over time [26,30,32], while some applied quantitative criteria (e.g., lesions enlarging by a certain volume or ≥12.5% per year on serial scans) [29,39], or required evidence of chronic active inflammation at the lesion edge (such as a paramagnetic rim) [37]. This heterogeneity reveals the lack of consensus on the standardized definition of SELs in the available literature [28]. More details on the principal characteristics of the included studies are presented in Table 1.

### 3.3. Data Synthesis

**Figure 2:**
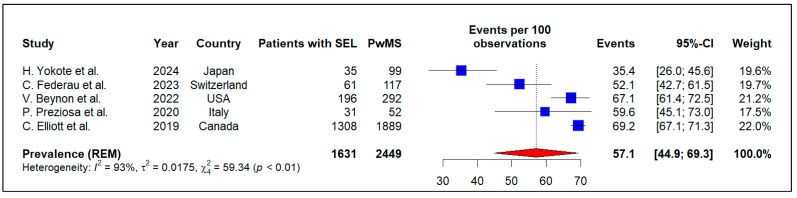
Forest plot of meta-analysis of the prevalence of slowly expanding lesions among people with multiple sclerosis [28,36,38,40,41].

A meta-analysis of five studies comprising 2449 PwMS estimated that the pooled prevalence of SEL in PwMS was 57.1% (95% CI: 44.9% to 69.3%, I^2^ = 93%, p-heterogeneity < 0.01) (Figure 2). Furthermore, Begg’s and Egger’s tests revealed no publication bias in this meta-analysis (*p*-value = 0.14 and *p*-value = 0.11, respectively).

### 3.4. Narrative Synthesis of Findings

According to the literature review, SELs appeared as an imaging biomarker that indicates chronic neuroinflammation, ongoing demyelination, and progressive tissue degeneration in MS [19,26,27,28,37,42]. Some cohort studies demonstrated that SEL was associated with an increased risk of disability progression, particularly in progressive MS phenotypes [19,29,32,33,38,40]. SELs also have relationships with microstructural damage and axonal degeneration that are characterized by reduced magnetization transfer ratios (MTR), increased radial diffusivity, and increased tissue destruction at lesion rims [28,30,39,42]. Several studies identified that paramagnetic iron rims and sustained microglial activity surround SELs [31,37,42]. These findings are aligned with the hypothesis that SELs reflect a smoldering, chronically active inflammatory process characteristic of progressive MS pathology.

Interventional and therapeutic studies have identified SELs as potential biomarkers that may reflect treatment response in MS. In clinical trials, patients treated with Bruton’s tyrosine kinase inhibitors (BTK) like Evobrutinib or the phosphodiesterase inhibitors like Ibudilast exhibited significantly less SEL volume compared with those receiving a placebo [17,27]. Retrospective studies demonstrated that using fingolimod [18] or natalizumab [36] led to fewer or smaller SELs, whereas ocrelizumab did not lead to a significant change in the frequency of SEL prevalence compared with placebo in primary progressive MS (PPMS) [29].

### 3.5. Risk of Bias Assessment

Table 1 summarizes the ROB scores for each study. Of the 20 studies, 12 achieved scores of 8 or higher (good quality), while the remaining scored between 6 and 7. The mean (SD) NOS score was 7.6 (0.8), indicating an overall moderate to high methodological quality (satisfactory) for the included studies.

## 4. Discussion

This systematic review and meta-analysis indicated that SELs are a common finding in PwMS, with an overall pooled prevalence of 57.1%. Evidence suggests that SELs can serve as important MRI indicators of chronic neuroinflammation, persistent demyelination, and gradual axonal loss. In addition, a higher SEL burden is linked to greater disability progression, particularly in PMS.

### 4.1. Pathobiology and Cellular Mechanisms of SELs in MS

SELs are chronic active, or smoldering lesions, that develop from earlier acute inflammatory sites [43,44]. Autopsy studies in PwMS revealed that 20% to 40% of WMLs are SELs, which are typically made up of CD8^+^ T and CD20^+^ B lymphocytes in the center, some plasma cells, a peripheral lattice of iron-laden activated microglia and macrophages, and proliferating oligodendrocytes at the border of the lesion [45,46]. Activated microglia and invading macrophages secrete pro-inflammatory cytokines and reactive oxygen species, causing chronic demyelination and axonal degeneration, while cytotoxic T cells cause direct neuronal injury [44]. On the other hand, B cells worsen the process through antibody secretion, cytokine release, and remyelination inhibition [8].

Over time, these lesions contribute to the clinical picture of progressive disease, marked by incomplete or failed remyelination, irreversible myelin loss, and, ultimately, poorer long-term outcomes such as increasing disability [19]. SELs are mostly identified through longitudinal MRI studies, particularly using repeated T1- and T2-weighted sequences that allow clinicians to observe their subtle and gradual expansion over time [38,45,47]. They occur independently of gadolinium enhancement, have less intense baseline T1, and progressively decrease in T1 signal, representing progressive axonal loss and chronic tissue injury [19]. Susceptibility-based MRI sequences reveal hypointense rims, similar to iron deposition in macrophages and microglia, near the lesion border, validating their classification as CALs [9]. This MRI finding is present in all MS phenotypes, but is more pronounced in PPMS [44].

### 4.2. Clinical Consequences of SELs in MS

A recent study found that rim lesions were present in about 9.8% of individual lesions and in 41% of patients. CALs were seen in 12% of lesions and in 64.5% of patients [39,48]. Studies using 7T MRI reported a higher proportion of patients with lesions compared with studies using 3T MRI. In contrast, older age and longer disease duration were linked to fewer lesions at the individual lesion level. This might likely reflect the transition of active lesions into chronic inactive scars over time. MRI sequence, gender, and EDSS did not significantly affect SEL prevalence [48]. In line with these findings, our meta-analysis demonstrated that SELs occur in 57.1% of PwMS, supporting their potential as an MRI marker of chronic lesion activity.

Recently, PRLs have been at the forefront of interest in MS research and have been included in the 2024 McDonald criteria as a supportive imaging biomarker to increase diagnostic specificity [3,12]. Their presence points to worse outcomes from disease aggressiveness and persistent lesion activity. Due to the high specificity of PRLs, they help to diagnose uncertain cases [12]. SELs are not currently included within the diagnostic criteria [14,15]. Considering the possibility of their explaining ongoing lesion activity and disease progression [38], SELs are being further investigated as an emerging biomarker in MS.

It currently appears that the dominant driver of disability accumulation in PwMS is progression independent of relapse activity (PIRA) [49], and SELs appear to be an important biological substrate of this silent progression. Longitudinal MRI studies demonstrate that SELs exhibit ongoing microstructural degeneration, including declining fractional anisotropy and progressive demyelination [26,30], and their presence is associated with higher risk of PIRA and faster disability worsening [32]. Linking SEL-related smoldering pathology with the insidious, relapse-independent disability accumulation characteristic of PIRA [26,30] supports SELs as a key imaging marker for identifying patients at risk of early and sustained progression.

### 4.3. SELs and Disease-Modifying Therapies in MS

Growing evidence showed that several disease-modifying therapies (DMTs) can influence SELs in PwMS. A clearer understanding of the inflammatory and cellular mechanisms underlying SEL development helps explain why many DMTs are capable of slowing or reducing their expansion [50]. BTKs, such as evobrutinib and tolebrutinib, are one of these DMTs. Evobrutinib reduces SEL volume by suppressing BTK activity in B cells and myeloid cells, which leads to limiting microglial activation [27]. Similarly, tolebrutinib has shown favorable long-term outcomes in reducing SEL volume in a 2-year extension study [51]. Ibudilast is a phosphodiesterase inhibitor and neuroprotective agent that reduced SEL volume by 23% and attenuated microglial and astrocytic activation [17]. Among established MS treatments, fingolimod is a sphingosine-1-phosphate receptor modulator that decreases peripheral lymphocyte trafficking and limits T-cell entry into the CNS. Treatment with fingolimod resulted in a reduction in the count and volume of SELs in PwMS [18]. Natalizumab, as an α4-integrin inhibitor, blocks immune-cell migration across the blood–brain barrier and mitigates chronic inflammation. It decreased the prevalence of SELs among PwMS in a longitudinal study [36]. Taken together, these findings highlight SELs as potential treatment-responsive biomarkers of active immune-mediated tissue damage in PwMS.

### 4.4. Prognostic Biomarkers in Multiple Sclerosis

CALs have been identified as significant prognostic imaging biomarkers in MS [10,38]. The current consensus definition of CALs encompasses PRLs, MRI-defined SELs, and TSPO-positive lesions on PET imaging, all of which represent ongoing inflammatory activity [9]. PRLs are characterized by more severe disease [52], brain and spinal cord atrophy [53], and a higher number of leptomeningeal enhancement foci [54]. Similarly, a higher SELs burden is predictive of the long-term progression of disability [38], greater EDSS and functional worsening [38], higher risk of secondary progressive MS conversion [38], and a higher proportion of persisting black holes [55]. Imaging data are also supported by molecular biomarkers [56]. Neurofilament light chain (NfL) represents axonal injury and is predictive of relapse risk, gadolinium-enhancing lesions, and confirmed disability worsening independent of relapses [56]. Another biomarker, glial fibrillary acidic protein (GFAP) is associated with grey matter atrophy, progression independent of relapse activity, and is correlated with SEL number [34,57].

CSF biomarkers further refine prognostic assessment, as the presence of oligoclonal bands (OCBs) predicts an increased risk of disability progression, approximately disability doubling the risk of disability progression [58], and are associated with relapses [59], cortical lesion burden [60], and secondary progression [60], which all reflect chronic B-cell-driven inflammation. Other biomarkers, such as parvalbumin [61], the kappa free light chain (KFLC) index [62], composite indices such as the CHI3L1*GFAP/NfL, known as “Glia score” [63,64], inflammatory cytokine patterns (e.g., CXCL13, IL-6, TNF, IFNγ) [65], mitochondrial DNA [66], and CSF lactate [67], have been associated with grey matter damage, aggressive disease course, lesion burden, and neurological deficits [66,67]. Altogether, SELs and PRLs define the anatomical basis of chronic inflammation [53], fluid biomarkers measure axonal damage [62], astroglial activation [68], and immune system dysregulation [68], and their joint evaluation could offer a comprehensive approach to prognostic stratification in MS.

## 5. Limitations and Directions for Future Research

This study has several limitations that should be acknowledged. First, as the meta-analysis was conducted on a limited number of eligible studies and substantial heterogeneity was observed among them, this limits the strength of the pooled estimates. Moreover, we were unable to perform meta-analyses on correlations or other secondary outcomes. The included studies varied widely in design, sample size, MRI field strength, acquisition parameters, segmentation techniques, and image analysis software, which may have contributed to variability in reported SEL frequencies and associated outcomes [52]. Second, the absence of susceptibility-weighted or T2-weighted imaging limited the ability to identify iron-laden or paramagnetic rim lesions, while the lack of post-contrast sequences prevented confirmation that baseline T2 lesions were pre-existing rather than newly active [37]. The relatively short follow-up period of approximately two years may also have been insufficient to fully capture the slow evolution and chronic activity of SELs across the included studies. Third, some analyses were based on small sample sizes, particularly for advanced imaging measures such as MTR, which means that Begg’s and Egger’s tests should be interpreted with caution. Quantification of SELs remains unstandardized, and inconsistencies in defining key features, such as lesion enlargement and concentric expansion, make it difficult to compare results between studies and reproduce findings. Technical variables such as differences between MRI scanners and analyzing software can compromise the accuracy of volumetric measurements, and the longitudinal interval was different among studies. In addition to methodological variability, clinical heterogeneity should also be considered when interpreting our findings. The pooled population had a spectrum of mild to moderate disability and included different MS phenotypes. Given that lesion dynamics and progression patterns may differ across disease stages and phenotypes, this variability may have influenced the observed prevalence estimates and limited the generalizability of our results. Future studies with large and multicenter studies that employ standardized imaging protocols, longer follow-up periods, and advanced MRI sequences are needed to explore the reliability of findings and improve the pathological and clinical understanding of SELs in MS.

## 6. Conclusions

In summary, current evidence suggests that SELs are present in about half of PwMS, with an overall prevalence of 57.1%. These lesions may be a potential radiomarker of chronic neuroinflammation, persistent demyelination, and progressive tissue loss. Additionally, SEL burden is linked to disability and disease progression, particularly in PMS. Future studies using standardized imaging protocols are needed to clarify the underlying immune mechanisms responsible for the enlargement of SELs and the effects of DMTs on their evolution to further investigate the relationships between SELs and various features in PwMS, confirming SELs as predictive biomarkers in MS.

## Figures and Tables

**Table 1:** The main characteristics of the included studies.

First Author	Year	Country	Study Design	Sample Size, F:M, Age; Mean (SD)	MS Subtype (*n*)	EDSS; Mean (SD)	Disease Duration (Years); Mean (SD)	MRI Device	Software of MRI Analysis	SEL Definition	Major Findings	QA
I. Vavasour [26]	2025	Canada	Cohort	50 31:19 40.1 (9.2)	RRMS: 50	2 (0–4.5) *	NR	Philips 3T	NR	Persistent, concentric expanding lesions.	SEL showed ongoing demyelination and MS progression.	8
A. Calvi [30]	2025	UK	Cohort	130 93:37 41.5 (34.6–49.2) **	RRMS: 117 PMS: 13	1.5 (1–2) **	6.8 (2.8–13.5) **	Siemens 3T	Jim 7	Lesions that gradually expand radially over time.	Definite SELs exhibit greater microstructural damage and are linked to PIRA.	8
D. L. Arnold [27]	2024	Canada	Cross-sectional	53 36:17 42.2 (11.5)	RRMS: 47 SPMS: 6	NR	NR	NR	NR	Progressive expansile T2 lesions detected on MRI.	NR	7
A. Cross [34]	2024	USA	Cohort	131 84:47 38.5 (9.6)	RRMS: 100 PPMS: 31	NR	NR	NR	NR	Lesions that gradually expand radially over time, representing chronic active lesions.	Higher SEL count is related to the elevation of Neurodegeneration.	8
H. Yokote [40]	2024	Japan	Cohort	99 72:27 40.2 (8.5)	NR	2 (1.7)	9.5 (7)	GE 3T	FreeSurfer 7.1.1 (Boston, MA, US)	Progressive expansile T2 lesions detected on MRI.	The number of SELs could be a biomarker of disease activity in PwMS.	8
K. Nakamura [17]	2024	USA	RCT	195 112:83 55.9 (7)	SPMS: 88 PPMS: 107	6 (4–6) **	NR	Siemens 3T	ANTs NR	Progressive expansile T2 lesions detected on MRI.	Ibudilast significantly reduced SEL volume.	8
A. Calvi [18]	2023	UK	Cohort	170 95:75 48 (27–64) *	PPMS: 170	4 (3–6.5) **	NR	Siemens 3T	Jim 7	Progressive expansile T2 lesions detected on MRI.	. Fingolimod treatment was linked to reduced SEL volume.	7
C. Federau [41]	2023	Switzerland	Cross-sectional	117 81:36 51.9 (11.7)	SPMS: 117	NR	NR	NR	Jazz NR	Preexisting white matter lesions showing expansion between prior and current examinations.	NR	7
S. Klistorner [42]	2023	Australia	Cohort	52 32:20 41.7 (9.1)	RRMS: 52	1 (0–4) *	5.5 (4.1)	GE 3T	Jim 9 (Xinapse Systems, Essex, UK)	Lesions reflecting ongoing low-grade demyelination activity.	Slow-burning inflammation at the lesion rim takes role in axonal irritation.	7
A. Calvi [31]	2022	UK	Cross-sectional	61 42:19 34.4 (14.1–64.9) *	RRMS: 61	1.5 (0–5.5) *	0.4 (0.1–16.6) *	Siemens 3T	Jim 7	Progressive expansile lesions detected on MRI.	Increasing SEL volume, indicating a possible link between chronic inflammation and global neuroaxonal damage.	8
P. Preziosa [38]	2022	Italy	Cohort	52 30:22 36.8 (9.7)	RRMS: 52	2 (1–4) **	9.8 (6.5)	Philips 3T	Jim 7	Lesions that gradually expand radially over time.	This study showed an increased risk of EDSS progression associated with SEL during follow-up.	7
M. Huerta [35]	2022	USA	Cohort	15 12:3 42.4 (5.6)	RRMS: 15	3 (1.5–3.5) **	8.5 (4.1)	Siemens 7T	In house method	Concentric expansile lesions over time on MRI.	PwMS with SELs showed more extensive demyelination than without one.	6
V. Beynon [36]	2022	USA	Cohort	600 NR	SPMS: 600	NR	NR	NR 3T	NR	Concentric expansile lesions over time on MRI.	Natalizumab reduced the prevalence of SELs.	8
A. Calvi [32]	2022	UK	Cohort	135 99:36 35.5 (9)	RRMS: 135	1.5 (0–5.5) **	5.5 (0–32.5) ^α^	Siemens 3T	Jim 7	Lesions that gradually expand over time.	Higher SEL volumes are associated with clinical progression, while lower ones are associated with stability in relapse-onset MS.	8
A. Calvi [33]	2022	UK	Cohort	345 230:115 55.9 (50–60.4) **	SPMS: 345	6 (5.5–6.5) **	21 (15–22) **	Siemens 3T	Jim 7	Progressive expansile lesions detected on MRI.	Definite SELs comprise about one-third of T2 lesions in SPMS.	8
C. Elliott [28]	2020	Canada	Cohort	299 194:105 NR	RRMS:242 SPMS:57	NR	NR	NR	NR	SELs lack a standardized definition across centers, limiting cross-study comparisons.	SELs exhibited reduced magnetization transfer ratios and increased radial diffusivity on DTR.	9
P. Preziosa [39]	2020	Italy	Cohort	52 30:22 36.8 (9.6)	RRMS: 52	NR	2.2 (1.7)	Philips 3T	Jim 7	Lesions with ≥10 voxels growing ≥12.5% annually over 2 years.	In PwMS, SELs showed reduced MTR and T1 signal intensity compared to non-SELs.	7
C. Elliott [19]	2019	Canada	Cohort	1889 1149:740 39.5 (8.8)	RRMS: 1334 PMS: 555	3.2 (1.3)	6.5 (5.3)	NR	NR	T2 lesions with slow, continuous outward expansion.	The number of SELs was greater in patients with PPMS.	9
C. Elliott [29]	2019	Canada	Cohort	555 276:279 45 (7.9)	PPMS: 555	4.6 (1.2)	6.1 (3.7)	NR	NR	Lesions with ≥10 voxels expanding ≥12.5% per year over 2 years.	The proportion of patients with “all SEL candidates” was similar between the ocrelizumab and placebo groups.	8
V. Sethi [37]	2016	USA	Cohort	22 14:8 32 (9)	RRMS:12 SPMS:10	NR	NR	GE Siemens Philips 3T 1.5T	Jim 7	SELs with minimal edge inflammation and microglial activity.	Gradual erosion of existing lesions alone does not fully explain tissue loss.	6

* Median (range), ** Median (IQR), ^α^ Mean (Range). ANTs: Advanced Normalization Tools, EDSS: Expanded Disability Status Scale, MS: Multiple sclerosis, MTR: Magnetization transfer ratio, PPMS: Primary progressive multiple sclerosis, RCT: randomized controlled trial, RRMS: Relapsing remitting multiple sclerosis, SEL: Slowly expanding lesions, SPMS: Secondary progressive multiple sclerosis, QA: Quality Assessment.

## Data Availability

All relevant data are within the paper and its Supporting Information files.

## Supplementary Materials

The following supporting information can be downloaded at: https://www.mdpi.com/article/10.3390/neurosci7020034/s1. Table S1: The searched syntax in each database.
