# Supplementary material for: Slowly Expanding Lesions in Multiple Sclerosis: A Systematic Review and Meta-Analysis
Source: NeuroSci. 2026 Mar 6;7(2):34. doi: 10.3390/neurosci7020034 (PMC13010691; doi:10.3390/neurosci7020034)
Supplement: Supplementary file 1 [file neurosci-07-00034-s001.zip › neurosci-4136606-supplementary.pdf]

Supplementary Material

Table S1. The searched syntax in each database.

Syntax	Results
PubMed/MEDLINE	
((sclerosis AND multiple) OR (sclerosis AND disseminated) OR "disseminated sclerosis" OR "multiple sclerosis" OR "acute fulminating")<br>AND<br>(“slowly expanding lesion” OR “slowly expanding lesions” OR (“slowly expanding” AND lesion) OR (slowly AND “expanding lesion”) OR “slowly evolving lesion” OR “slowly evolving lesions” OR (“slowly evolving” AND lesion) OR (slowly AND “evolving lesions”) OR “smoldering lesion” OR “chronic active lesion” OR (“chronic active” AND lesion) OR (chronic AND “active lesion”) OR “chronic smoldering lesion” OR (“chronic smoldering” AND lesion) OR (chronic AND “smoldering lesion”) OR “lesion expansion” OR “slowly enlarging lesion” OR (“slowly enlarging” AND lesion) OR (slowly AND “enlarging lesion”) OR “iron rim lesion” OR (“iron rim” AND lesion) OR (iron AND “rim lesion”) OR “rim-positive lesion” OR “paramagnetic rim lesion” OR (“paramagnetic rim” AND lesion) OR (paramagnetic AND “rim lesion”) OR “rim-negative lesion” OR “non-enhancing chronic lesion” OR (“non-enhancing chronic” AND lesion) OR (non-enhancing AND “chronic lesion”) OR “MRI-defined SEL” OR “persistent black hole” OR (“persistent black” AND hole) OR (persistent AND “black hole”))	476
Scopus	
TITLE-ABS((sclerosis AND multiple) OR (sclerosis AND disseminated) OR "disseminated sclerosis" OR "multiple sclerosis" OR "acute fulminating")<br>AND<br>TITLE-ABS(“slowly expanding lesion” OR “slowly expanding lesions” OR (“slowly expanding” AND lesion) OR (slowly AND “expanding lesion”) OR “slowly evolving lesion” OR “slowly evolving lesions” OR (“slowly evolving” AND lesion) OR (slowly AND “evolving lesions”) OR “smoldering lesion” OR “chronic active lesion” OR (“chronic active” AND lesion) OR (chronic AND “active lesion”) OR “chronic smoldering lesion” OR (“chronic smoldering” AND lesion) OR (chronic AND “smoldering lesion”) OR “lesion expansion” OR “slowly enlarging lesion” OR (“slowly enlarging” AND lesion) OR (slowly AND “enlarging lesion”) OR “iron rim lesion” OR (“iron rim” AND lesion) OR (iron AND “rim lesion”) OR “rim-positive lesion” OR “paramagnetic rim lesion” OR (“paramagnetic rim” AND lesion) OR (paramagnetic AND “rim lesion”) OR “rim-negative lesion” OR “non-enhancing chronic lesion” OR (“non-enhancing chronic” AND lesion) OR (non-enhancing AND “chronic lesion”) OR “MRI-defined SEL” OR “persistent black hole” OR (“persistent black” AND hole) OR (persistent AND “black hole”))	559
Web of Science	

ALL=((sclerosis AND multiple) OR (sclerosis AND disseminated) OR "disseminated sclerosis" OR "multiple sclerosis" OR "acute fulminating")<br>AND<br>ALL=("slowly expanding lesion" OR ("slowly expanding" AND lesion) OR (slowly AND "expanding lesion") OR "slowly evolving lesion" OR ("slowly evolving" AND lesion) OR (slowly AND "evolving lesions") OR "smoldering lesion" OR "chronic active lesion" OR ("chronic active" AND lesion) OR (chronic AND "active lesion") OR "chronic smoldering lesion" OR ("chronic smoldering" AND lesion) OR (chronic AND "smoldering lesion") OR "lesion expansion" OR "slowly enlarging lesion" OR ("slowly enlarging" AND lesion) OR (slowly AND "enlarging lesion") OR "iron rim lesion" OR ("iron rim" AND lesion) OR (iron AND "rim lesion") OR "rim-positive lesion" OR "paramagnetic rim lesion" OR ("paramagnetic rim" AND lesion) OR (paramagnetic AND "rim lesion") OR "rim-negative lesion" OR "non-enhancing chronic lesion" OR ("non-enhancing chronic" AND lesion) OR (non-enhancing AND "chronic lesion") OR "MRI-defined SEL" OR "persistent black hole" OR ("persistent black" AND hole) OR (persistent AND "black hole"))	647
Embase	
((sclerosis AND multiple) OR (sclerosis AND disseminated) OR 'disseminated sclerosis' OR 'multiple sclerosis' OR 'acute fulminating')<br>AND<br>('slowly expanding lesion' OR 'slowly expanding lesions' OR ('slowly expanding' AND lesion) OR (slowly AND 'expanding lesion') OR 'slowly evolving lesion' OR 'slowly evolving lesions' OR ('slowly evolving' AND lesion) OR (slowly AND 'evolving lesions') OR 'smoldering lesion' OR 'chronic active lesion' OR ('chronic active' AND lesion) OR (chronic AND 'active lesion') OR 'chronic smoldering lesion' OR ('chronic smoldering' AND lesion) OR (chronic AND 'smoldering lesion') OR 'lesion expansion' OR 'slowly enlarging lesion' OR ('slowly enlarging' AND lesion) OR (slowly AND 'enlarging lesion') OR 'iron rim lesion' OR ('iron rim' AND lesion) OR (iron AND 'rim lesion') OR 'rim-positive lesion' OR 'paramagnetic rim lesion' OR ('paramagnetic rim' AND lesion) OR (paramagnetic AND 'rim lesion') OR 'rim-negative lesion' OR 'non-enhancing chronic lesion' OR ('non-enhancing chronic' AND lesion) OR (non-enhancing AND 'chronic lesion') OR 'MRI-defined SEL' OR 'persistent black hole' OR ('persistent black' AND hole) OR (persistent AND 'black hole'))	701
